# DNMSO; an ontology for representing de novo sequencing results from Tandem-MS data

**DOI:** 10.7717/peerj.10216

**Published:** 2020-10-21

**Authors:** Savaş Takan, Jens Allmer

**Affiliations:** 1 Department of Computer Engineering, Faculty of Engineering, Izmir Institute of Technology, Izmir, Turkey; 2 Hochschule Ruhr West, University of Applied Sciences, Medical Informatics and Bioinformatics, Institute for Measurement Engineering and Sensor Technology, Mülheim an der Ruhr, Germany

**Keywords:** Mass spectrometry, De novo sequencing, Ontology, DNMSO, DNML, Format

## Abstract

For the identification and sequencing of proteins, mass spectrometry (MS) has become the tool of choice and, as such, drives proteomics. MS/MS spectra need to be assigned a peptide sequence for which two strategies exist. Either database search or de novo sequencing can be employed to establish peptide spectrum matches. For database search, mzIdentML is the current community standard for data representation. There is no community standard for representing de novo sequencing results, but we previously proposed the de novo markup language (DNML). At the moment, each de novo sequencing solution uses different data representation, complicating downstream data integration, which is crucial since ensemble predictions may be more useful than predictions of a single tool. We here propose the de novo MS Ontology (DNMSO), which can, for example, provide many-to-many mappings between spectra and peptide predictions. Additionally, an application programming interface (API) that supports any file operation necessary for de novo sequencing from spectra input to reading, writing, creating, of the DNMSO format, as well as conversion from many other file formats, has been implemented. This API removes all overhead from the production of de novo sequencing tools and allows developers to concentrate on algorithm development completely. We make the API and formal descriptions of the format freely available at https://github.com/savastakan/dnmso.

## Introduction

Proteomics is the study of proteins, and one popular tool in proteomics is mass spectrometry (MS), which is its driving force and has applications for protein identification, sequencing, and quantitation. Mass spectrometry measures the mass to charge ratios (m/z) of peptides, but following fragmentation of the peptide precursors an additional stage of MS (MS/MS, tandem-MS, *MS*^2^) can establish the m/z of the resulting fragments. The resulting MS/MS spectra can be assigned a peptide, and the resulting peptide spectrum match (PSM) can be used for further analysis, such as protein identification and sequencing. Two approaches are possible to assign a PSM, one that needs a database of expected sequences or a library of identified spectra (database search) and one which directly determines the peptide sequence from the MS/MS spectrum (de novo sequencing). Many algorithms are available for database search (Hoopmann & Moritz, 2013) and de novo sequencing (Allmer, 2011). It might appear that with the availability of many sequenced genomes, the need for de novo sequencing diminishes. Still, for several reasons (Allmer et al., 2004; Reboul et al., 2003; Medzihradszky & Chalkley, 2015), such as alternative splicing and post-translational modifications, this is unlikely.

A variety of different mass spectrometers are available from a multitude of vendors, and all mass spectra are represented in some proprietary format, which complicates the sharing of data. This problem has been tackled by proposing an open standard representation for such data, and an early example for the representation of MS and *MS*^*n*^ spectra is mzXML (Pedrioli et al., 2004). Today the human proteome Organization (HUPO) is overseeing the standardization, and the latest mass spectra representation standard is mzML (Deutsch, 2010; Martens et al., 2011). There are, however, many other formats such as dta, mgf, and pkl which are widely used despite the available standard. Every algorithm that predicts PSMs needs mass spectra as input and thus depends on one or more of such file formats or standards.

On the other hand, each algorithm that assigns PSMs needs to represent the results in some way. There are many vendor-specific representations, but in this area, an early push for standardization was pepXML and protXML (Keller et al., 2005). The currently supported standard by HUPO is mzIdentML (Jones et al., 2012). The results include, for example, links to the MS/MS spectrum supporting a PSM and to the database containing the peptide. De novo sequencing employs no database, and we further identified parts of the mzIdentML and pepXML standards as unnecessary for the representation of de novo sequencing results. For instance, sample handling and sample collections should be the concern of a different standard, modeling experiments. A standard should be targeted and lean so that representation is not unnecessarily inflated and does not contain needless fields. Besides, presenting de novo sequencing results in pepXML format, as PEAKS (Ma et al., 2003) can do, may confuse downstream analysis since a database search standard representation is produced while de novo sequencing was performed. Finally, Creasy and colleagues specifically encouraged to help to represent de novo sequencing results (Jones et al., 2012), and Field & Sansone (2006) generally encourage standard propositions from the community since they foster dialogue and raise awareness while opening roads to actions. Although Creasy and colleagues likely intended representation of de novo results within mzIdentML, we believe that it is better to separate database search and de novo sequencing result standards. While both approaches are very related, de novo sequencing is the general case of database search where the database includes all possible peptides, the intention of database search is the identification of peptides and proteins. In contrast, the aim of de novo sequencing is—sequencing, which may or may not lead to an unambiguous identification.

We previously proposed a format for the representation of de novo sequencing results (DNML) (Takan & Allmer, 2012) based on XML, just like all standards introduced above. The extensible markup language, used in our and the previously mentioned standards, is inherently based on a tree representation and is therefore not the best representation for networks, which is the native format of the data. New technological advances allow the representation of data in an ontology format, which is inherently network-based (Bodenreider & Stevens, 2006) and, therefore, better suited to represent the results of database search and de novo sequencing algorithms. We realized this blemish of our DNML format and set forth to develop an ontology-based representation for de novo sequencing results.

In this study, we present the de novo MS ontology (DNMSO) format as a proposition for a standard representation of de novo sequencing results. The format is entirely based on ontology representation, which is easily extensible and adaptable in response to community requests. We further include the PSI-MS (Mayer et al., 2013) controlled vocabulary and the PSI-MOD (Montecchi-Palazzi et al., 2008) post-translational modifications (PTMs) ontology in DNMSO. We believe that providing an application programming interface (API) is essential for acceptance of a format as a standard in the community. It is not enough to provide an interface for reading, but writing and creating files in the format must be supported. In addition to these requirements, our API further supports reading of spectra in many common and standard file formats as well as conversion from pepXML, DNML, and many other formats like Lutefisk (Taylor & Johnson, 1997) results and PepNovo (Frank & Pevzner, 2005) results into DNMSO format. With this API, we hope to ensure that developers of new de novo sequencing algorithms can fully concentrate on algorithm development and can rely on our API for file handling. Tools that automate *de novo* sequencing algorithms such as DeNovoGUI (Muth et al., 2014) would benefit from having to deal with only one file format and could generalize their tool to incorporate more than just PepNovo+ (Frank & Pevzner, 2005). A multitude (>20) of de novo sequencing tools have been proposed (Muth et al., 2016) with three additions in 2019 (Yang et al., 2019; Azari et al., 2019; Tran et al., 2019). The de novo sequencing community would benefit from a standard file format for data sharing and data integration.

## de novo MS Ontology

Ontologies, such as Gene Ontology (Carbon et al., 2019), are used to capture knowledge about a domain of interest, in this case, de novo sequencing. An ontology describes the concepts in the domain and also the relationships that hold among them (Alterovitz et al., 2010). Different ontology languages provide different facilities. OWL, from the World Wide Web Consortium (W3C), is an ontology modeling language, including a rich set of operators such as intersection, union, and negation. It is based on a logical model, which makes it possible for concepts to be described. Complex concepts can, therefore, be built up of simpler concepts (McGuinness & Van Harmelen, 2004). The TopBraid Composer ME was used as the ontology editor to create ontology relationships and structure. The complete ontology, descriptions, annotations, specifications, and the API are available in our GitHub repository: https://github.com/savastakan/dnmso.

**Figure 1:**
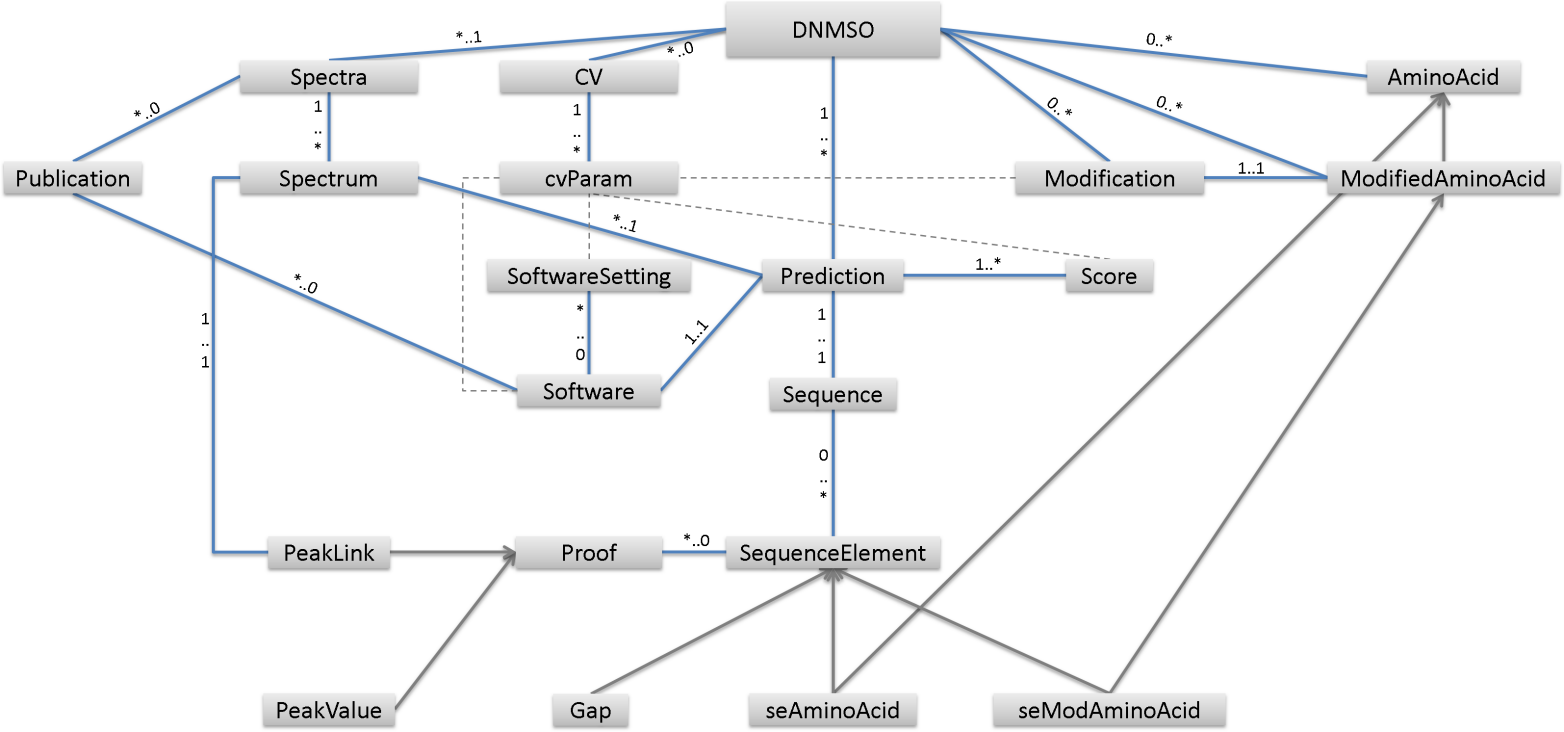
Displayed are the high-level elements in the DNMSO ontology. Elements are connected by blue lines, which show the multiplicity of elements for each other. Gray arrows indicate inheritance, with the element, pointed to being the base element. Dashed lines indicate other links among elements (e.g., the use of cvParams).

A high-level overview of the proposed ontology is given in Fig. 1, and additional detail is provided in our GitHub repository. Many specifics, such as what exactly needs to be known about the Software element, are not provided in Fig. 1 but can be further investigated by exploring or downloading the ontology from our GitHub page (https://savastakan.github.io/dnmso/). Each PSM needs at least one peptide prediction and one MS/MS spectrum. Figure 1 shows that the DNMSO ontology has Prediction and Spectra as well as Modification elements. Prediction models the PSM by providing a Sequence, sources (at least one Spectrum), and at least one Score. A source is modeled via a link to the Spectrum, which is contained in the file or linked from other files. This model entails that at least all spectra, which lead to a prediction, must be provided in any instance of the DNMSO format.

It is permissible to store spectra that were not used, i.e., have no predictions, but it is not allowed to create predictions without associated spectra. Currently, there are two means to represent spectra, either through a link to a file containing the spectrum, for example, a file in mzML format. Since most spectra are usually not successfully assigned a peptide, this means that for data sharing, the full, often large, file containing the raw data needs to be distributed along with the results. To simplify data exchange, we also allow the spectra to be represented in CSV format so that all necessary raw data can be contained in the DNMSO format in such instances. Allowing to include all necessary data in an instance, ensures minimum file size and guarantees that if the DNMSO file is present, all raw data used to create it is also contained in the instance.

To ensure that DNMSO can be used standalone, post-translational modification information is stored in the instances. However, each PTM must provide a reference to a PSI-MOD term. The DNMSO instances only store the PTMs that were used/identified and does not incorporate the many PTMs available in PSI-MOD.

A Prediction represents the actual PSM, but several predictions even by the same de novo sequencing software, using the same settings, can be represented in DNMSO as separate Prediction elements. A Prediction has one or more associated scores for the PSM. Each PSM can be associated with multiple spectra, which is essential with the advent of tools that use multiple spectra for the assignment of a PSM. For instance, PepNovo+ (Frank, 2009) merges multiple spectra into one, when possible, which can be represented as a spectrum in CSV format or if a trivial merging was performed by linking all merged spectra as sources. Other recent algorithms combine multiple spectra from the same precursor after fragmentation by different methods, for example, collision-induced dissociation (Wells & McLuckey, 2005) (CID) and electron transfer dissociation (Syka et al., 2004) (ETD). SpectrumFusion (a de novo sequencing algorithm) uses CID and ETD spectra of the same precursor to making a de novo prediction (Datta & Bern, 2009). Merged spectra cannot be considered raw data anymore, so our format needs to be able to account for that, and we modeled it by allowing any number of spectra to be associated with a peptide prediction. Therefore, not the merged spectra need to be represented in a new mzML or mzXML file, but each Prediction element needs to be associated with all the spectra that were used for the prediction. Alternatively, they can be represented in CSV format if the merging process discards peaks or introduces some changes to the peaks in the spectrum. Finally, any spectrum can be part of many predictions so that a many-to-many relationship (Prediction – Spectrum) needs to be modeled, which is achievable in XML. However, the solutions can be awkward, and thus this was another reason for us to choose an ontology over an XML representation. Moreover, XML does not provide semantics, and despite many XML approaches to include semantics in biology, a more radical solution is to use ontologies directly (Antezana, Kuiper & Mironov, 2009).

Representing the sequence could be thought of as straightforward, but that is hardly the case. We have seen many ways to represent PTMs, for instance, by using symbols that are not from the set of amino acids. This practice is limiting since, in any subsequent sequence search (e.g., BLAST (Altschul et al., 1990)), the amino acid must be known, and the PTM is irrelevant. However, then it becomes nontrivial to determine the amino acid underlying, for example, @. In our model, a sequence is made up of SequenceElements, which can be seAminoAcid, seModifiedAminoAcid, or Gap. A seModifiedAminoAcid reduces to an AminoAcid but also has the Modification information. Since some de novo sequencing algorithms do not produce full-length sequences, at times, it is important to allow the representation of a mass gap in the sequence. Currently, there are different ways to do that, for example, giving the unexplained mass in parentheses. In our format, a sequence is formed of these three elements. We believe that all sequence predictions can be modeled in this way. In our opinion, this is also useful since confidence can be attached to each sequence element. At the same time, proofs (supporting peaks) can be provided for each Sequence Element.

We have deliberately chosen not to include any information about sample handling and other experimental procedures since the information about the particular measurements are to be provided in the referenced mzML format or some other experiment standard. In concordance with good computer engineering design principles, information about experimental procedures should be provided in a targeted ontology such as described by Morrison and colleagues (Morrison et al., 2006). Therefore, DNMSO is only modeling its responsibilities. Including too many responsibilities in one model can cause problems. For example, analysisXML (Orchard et al., 2008) was later split into mzIdentML and mzQuantML (Walzer et al., 2013). Ball and Brazma also point out that restriction of scope is important and showcase this by their decision only to describe microarray data in their consortium (Ball & Brazma, 2006).

DNMSO does not exist in a void, and there are complementary standards and standards used within, as well as one target standard. We support mzML (default), mzXML standards (as well as mgf and some other file formats) to populate an instance of DNMSO with mass spectrometric data. PSI-MOD is the standard of choice for us to link to when defining modifications, and PSI-MS is the controlled vocabulary we intend to use in DNMSO. DNMSO can be converted from pepXML and other file formats such as PepNovo and Lutefisk. The final output could be an mzIdentML file after running de novo sequencing using multiple tools, applying a reasoner, associating with protein information (where available), and finally exporting to mzIdentML using the API provided.

## Application Programming Interface

Proposing a formal representation is beneficial to a field because, usually, a multitude of software collaborate to achieve a research goal. For de novo sequencing, examples for downstream analysis could be a visualization of low confident (but interesting) results or assembly of predicted peptide sequences into proteins. The use of ensemble or consensus predictions would also benefit from a standard format. In DNMSO, this can be achieved by writing SPARQL queries that combine multiple predictions into a consensus prediction. This consensus prediction can then again be stored in the DNMSO format.

We believe, however, that providing a formal representation is not sufficient and that it is essential also to provide all functionality to handle the proposed format computationally. This need for an API is because re-implementations of the format by different researchers or vendors inevitably lead to errors that reduce the usefulness of the format. Logical errors can also occur if the format is misinterpreted. Providing one single interface to handle the format by the developers of the format is, in our opinion, the best solution.

**Figure 2:**
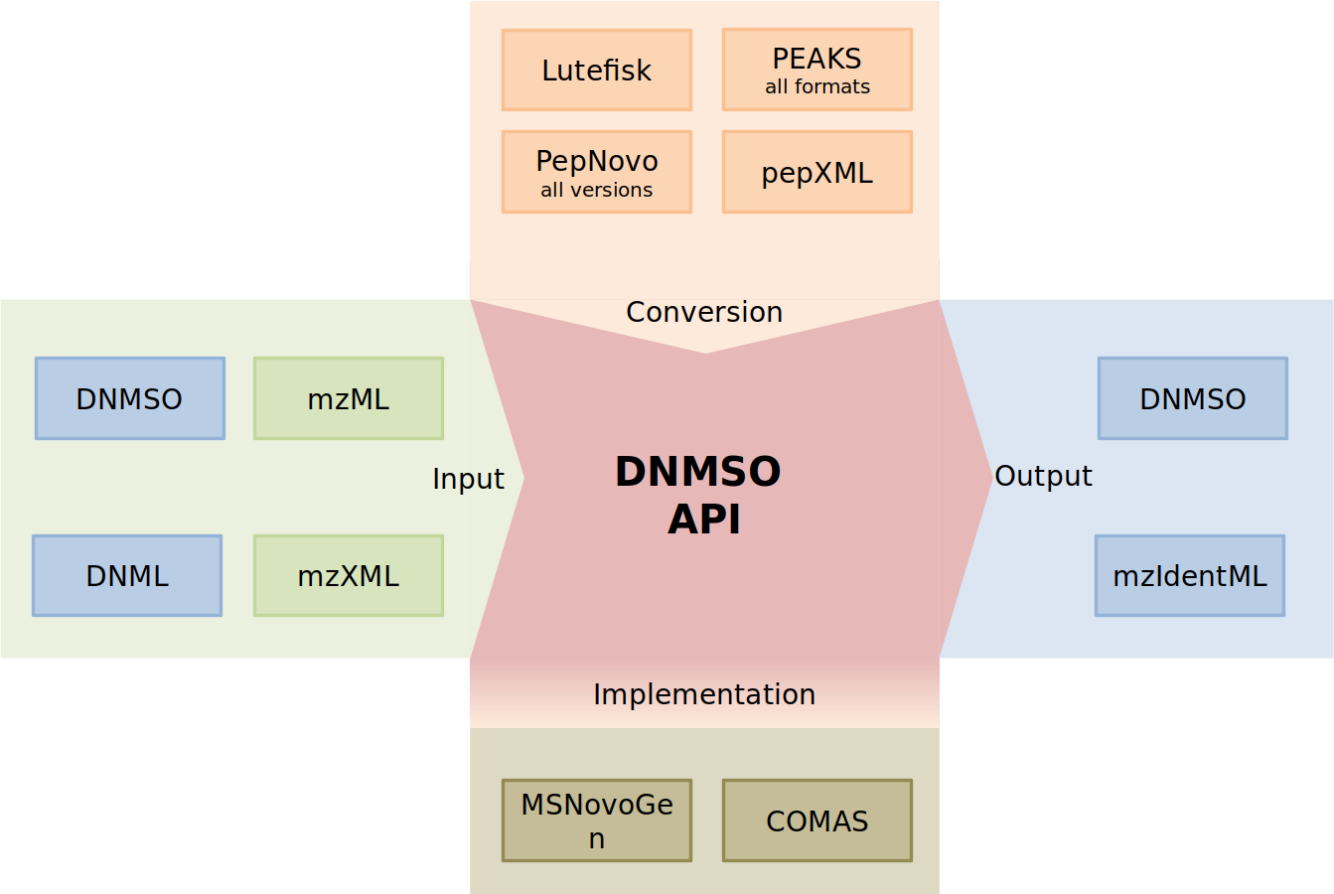
Depiction of the DNMSO API (red). MS/MS Spectra (green on green) and the DNML and DNMSO formats (blue on green) can be read using the DNMSO API. The API can convert existing formats (orange). The API is called in some in-house de novo sequencing algorithms (olive). Two possible outputs can be generated (blue).

The DNMSO API provides all functionality necessary to handle the format and offers additional functionality (Fig. 2).

We acknowledge that de novo sequencing has been performed for a long time and that the results that were produced in the last decades should not be voided. Therefore, we implemented the DNMSO API such that it is easily extensible and makes it trivial to add new conversion facilities. Currently, we support all PepNovo versions, all PEAKS outputs, Lutefisk, DNML (not shown), and pepXML conversion to our DNMSO format. We will implement further conversion facilities, but on the website, we also explain how a conversion module can be implemented and can be integrated without rebuilding our API.

For new de novo sequencing algorithms that opt to use the DNMSO API, we conveniently build in reading support for the latest two standard formats (mzXML and mzML). We implement reading support so that related standards do not need to be learned and that the developer of a new de novo sequencing approach can completely concentrate on the algorithm development. In case other spectra formats must be read, we allow runtime binding of such readers and explain on our website how that can be implemented without rebuilding the API. For any downstream analysis tool which will use the API, we also support reading of the DNMSO and DNML formats.

The most important part, which in our experience is missing from all so far proposed standards, is the creation of files in the proposed format. In Fig. 2, the part labeled implementation indicates the ability of a third-party tool to rely on the library to handle all data representation for the DNMSO format in memory and to write it to file on request. We confirmed this ability with two unpublished in-house tools (COMAS and MSNovoGen).

Finally, the DNMSO API provides the functionality to write the DNMSO format. How the data was filled into the API object (by reading, by creating data through an algorithm, or through conversion) is irrelevant to the writing facility.

The DNMSO API has been implemented using JAVA™. The RDF format, representing the ontology, is handled by the Jena Library. The JmzReader, GUI version 1.2.7 (http://code.google.com/p/jmzreader/), is used for reading mzXML and mzML data. Providing a JAVA API is sufficient since most programming languages can bind JAVA libraries. As an example, we provide Python code to show how to bind our JAVA API to create a simple file in DNMSO format (https://github.com/savastakan/dnmso), but a similar approach is possible for C, C++, R, and JavaScript (server-side and client-side). The JAVA API is also useful as a reference implementation of the DNMSO format; however, as it can be directly called from all popular languages in bioinformatics (except for Perl), the need to implement a custom library for handling the DNMSO format should not arise.

## Comparison to other Formats

Most de novo sequencing algorithms use *ad hoc* formats to represent their results, and we do not find it instructive to dissect them here. However, we do represent them on our website, which provides further information on the DNMSO format (https://github.com/savastakan/dnmso). We further provide a conversion facility for several output formats of existing de novo sequencing algorithms with the API.

Our previous format (DNML) was built on extensible markup language (XML), which is inherently tree-based. In de novo sequencing, however, predictions and spectra form a many-to-many relationship, something that is awkward to model in XML (http://www.ibm.com/developerworks/library/x-xdm2m.html).

Furthermore, the ontology model allows the use of a reasoner (Wang et al., 2004), which can ensure that statements and definitions are mutually consistent and can also recognize whether concepts fit under the given definitions. Apart from being cutting edge technology, ontologies have many advantages, such as high modularity.

DNMSO, the work presented here, is not only porting DNML to a novel and more modern format but also includes enhancements. For instance, although it is possible to represent multiple spectra for one result in DNML, the merging of spectra is not supported. DNMSO allows the merging of many spectra, something that is, for instance, done by PepNovo+ and allows the representation in CSV format. DNML allowed only one score, but many programs, such as Lutefisk, calculate multiple scores, and they are representable in DNMSO.

PEAKS, a commercial de novo sequencing tool, uses pepXML to represent its results. We support its use of a standard representation. However, we disagree with the usage of a database search format in the de novo sequencing realm, which may lead to confusion in downstream analyses. Furthermore, the current community standard to represent PSMs considering database search results is mzIdentML, but PEAKS employs the pepXML format. Tools that analyze data from database search engines may, for instance, be confused why no database is presented in the results. Additionally, if that is ignored, the results may be viewed from a database search result confidence and not from a de novo sequencing confidence so that they may be misinterpreted. In short, de novo sequencing needs its own standard representation and ought not to use a standard from a different even if strongly related domain.

Since pepXML is used to represent de novo predictions, we decided to briefly comment on some of the differences between the database search and de novo sequencing domains (Table 1). Furthermore, some of our comments may apply to the database search domain as well, and it may be beneficial to implement such notions in future versions of mzIdentML. Naturally, de novo sequencing does not need to represent any information about a database that was searched since the database searched in de novo sequencing consists of all peptides matching the precursor mass with a given mass error. In database search, it is necessary to provide the database source, its version, and many other parameters as well as proteins that match a given MS/MS spectrum. For database search, these parameters are essential and should be mandatory in the standard, which would *a priori* exclude de novo sequencing tools from using such formats. The mzIdentML community standard has weakened its requirement for associating a protein with a PSM in an attempt to make it useful for de novo sequencing (Vizcaíno et al., 2017). Nevertheless, PEAKS is still using pepXML for the representation of de novo sequencing results in 2020 while it represents SPIDER search results in mzIdentML format.

**Table 1:** Comparison of some features of different file formats for de novo sequencing.

	DNMSO	DNML	LutefiskXP	PepNovo	pepXML	mzIdentML
type	Ontology + CV	XML	flat	flat	XML	XML + CV
self-contained	+	+	–	–	–	–
Conversion to DNMSO		yes	yes	yes	yes	Not yet
Conversion from DNMSO		lossy	lossy	lossy	lossy	lossy
manipulation	full	full	partial	partial	partial	full
size	small	small	small	small	larger	larger
extensibility	yes	yes	no	no	yes	yes
artificial intelligence facilities	Yes, Logic reasoning	no	no	no	no	no
SPARQL	yes	no	no	no	no	no
Main aim	De novo sequencing	De novo sequencing	De novo sequencing	De novo sequencing	Peptide identification	Protein identification
Communication with other ontologies	Yes (PSI-CV, PSI-MOD)	no	no	no	no	Yes (PSI-CV, PSI-MOD)

We believe that all de novo sequencing results should be represented in the DNMSO instance but acknowledge that this may lead to large file sizes. Therefore, similarly to the approach taken by mzIdentML, we allow a threshold to be specified for any or multiple scores in the Prediction element of our ontology. We extend upon mzIdentML by allowing multiple scores and by allowing larger, lesser, and equal constraints on them for filtering. Filtering for DNMSO would mean constructing SPARQL queries.

Despite our analysis of pepXML and mzIdentML, it is not clear to us whether multiple mass spectra from different sources (e.g., CID and ETD fragmentation) can be used in tandem for database search. It is our current assumption that it is not possible. DNMSO supports this notion since it has been reportedly used in de novo sequencing (Allmer, 2011).

The database search standards offer confidence on the PSM level or if a decoy strategy is employed on the population level. On the PSM level, mzIdentML allows defining a set of peaks that supports the conclusion. DNMSO expands on this by allowing a set of peaks to support each element of the sequence, which may be gaps, amino acids, or modified amino acids. This setup also allows modeling ambiguity at the termini of peptide predictions stemming from the fact that MS/MS spectra are usually not well populated at the two extremes. This problem also affects database search algorithms, and it may be beneficial to provide not only PSM confidence but amino acid confidence as well for the mzIdentML standard.

Terminal peptide and amino acid modifications must be supported in both models. PSI-MOD (Montecchi-Palazzi et al., 2008) is an ontology to represent such modifications, and DNMSO requires a reference to PSI-MOD for each modification used in an instance. The DNMSO format records additional information such as average and monoisotopic masses to enable standalone distribution of DNMSO instances. The DNMSO API can read PSI-MOD and match its modifications to modifications represented in the currently supported de novo tool formats and allows the selection of modifications from PSI-MOD for file creation.

## Conclusions

De novo sequencing (Allmer, 2011), although not highly accurate, is gaining importance and interest since it supports the detection of novel peptides, which may not be annotated in protein databases. Unfortunately, much attention is diverted to unnecessary file handling, which could be used to improve de novo sequencing algorithms. We removed the input file handling problem by providing an API that allows developers to read spectra from the two most important standards, modeling mass spectra, mzXML, and mzML. We removed the output file handling problem by developing an ontology, DNMSO, and providing an API to read, write, and create files in the format. The availability of an API speeds up the development of future de novo sequencing algorithms. It will spawn the development of tools that build on de novo prediction such as the GenomicPeptideFinder (Allmer et al., 2004).

DNMSO is enhanced with an API by including further ontologies in addition to the PSI-MOD ontology. We have restricted possible values for most fields in the DNMSO format by using a custom ontology. We also include support for the PSI-MS controlled vocabulary, although it currently lacks relevant terms. However, once a community builds around DNMSO, PSI-MS is likely to include de novo sequencing specific terms. Independently from this, we have provided further conversion functionality for de novo sequencing tools.

Field and Sansone caution that it is difficult to gain acceptance for a standard due to technological or sociological reasons (Field & Sansone, 2006). We believe having solved the technological part by providing a fully-featured API. We do not expect any sociological problems, and we plan to offset resistance to use our API by providing further conversion facilities for the 20+ existing de novo sequencing tools.

Additional information, including tutorials and documentation as well as the API and ontology description, are available at https://github.com/savastakan/dnmso.

## Additional Information and Declarations

### Data Availability

The following information was supplied regarding data availability: Data and code are available at Github: Savaş Takan and Jens Allmer (2020). ”DNMSO; an Ontology for Representing De Novo Sequencing Results from Tandem-MS Data”. Github. Bioinformatics tool and data. https://github.com/savastakan/dnmso.
